# Differential Susceptibility to Heavy-Metal–Induced Injury in a Human Testis Tissue Equivalent (hTTE): Implications for Oxidative Stress

**DOI:** 10.3390/antiox15101311

**Published:** 2026-10-12

**Authors:** Adam B. Cohen, Janmejay Hingu, Abinav Udaiyar, Nima Pourhabibi Zarandi, Banafsheh Nikmehr, Omar A. Abdelaal, Nicos Prokopiou, Samuel Pendergraft, Anthony Atala, Colin E. Bishop, Stephen J. Walker, Hooman Sadri-Ardekani

**Affiliations:** 1 Wake Forest Institute of Regenerative Medicine, 391 Technology Way NE, Winston-Salem, NC 27101, USA; cohena10@ccf.org (A.B.C.); janmejay.hingu@advocatehealth.org (J.H.); abinav.udaiyar@advocatehealth.org (A.U.); nima.pourhabibizarandi@vcuhealth.org (N.P.Z.); bnikmehr@cfi.clinic (B.N.); omarelayman88@gmail.com (O.A.A.); nicos.prokopiou@advocatehealth.org (N.P.); samuel.pendergraft@wfusm.edu (S.P.); colin.bishop@wfusm.edu (C.E.B.); stephen.j.walker@advocatehealth.org (S.J.W.); 2 Department of Urology, Atrium Health Wake Forest Baptist, 1 Medical Center Boulevard, Winston-Salem, NC 27157, USA

**Keywords:** male infertility, oxidative stress, reactive oxygen species, reactive nitrogen species, heavy metals, mercury, lead, thallium, testicular hTTEs, spermatogonia, Sertoli cells, Leydig cells, antioxidants, microRNA, reproductive toxicology

## Abstract

Oxidative stress driven by environmental and occupational exposure to heavy metals is increasingly recognized as a central, modifiable contributor to male infertility. Yet, the lack of physiologically relevant human models has limited mechanistic insight. We have previously described a three-dimensional Human Testis Tissue Equivalent (hTTE) that recapitulates the cellular composition and stem cell niche of the seminiferous tubule and is more resistant than two-dimensional (2D) culture to gonadotoxic chemotherapeutics. Here, we used the hTTE platform to compare heavy-metal cytotoxicity, cell-type–associated transcript responses, and exploratory microRNA (miRNA) profiles elicited by three prototypical pro-oxidant heavy metals: mercury (Hg^2+^), thallium (Tl^+^), and lead (Pb^2+^). Background and Aim: To determine whether the hTTE model exhibits greater resistance to heavy-metal cytotoxicity than matched 2D cultures and whether cell-type–associated responses differ following heavy-metal exposure within the established redox biology of these toxicants. Methods: Adult human testicular cells from three donors were expanded, characterized by RT-qPCR and flow cytometry, and assembled into hTTEs alongside matched 2D cultures. Cultures were exposed for 48 h to Hg^2+^, Tl^+^, or Pb^2+^ across graded concentrations; IC_50_ values were derived from ATP-based viability assays and used for selected downstream exposure concentrations. The relative abundances of cell-type–associated transcripts were profiled by digital PCR for ZBTB16 (spermatogonia), STAR (Leydig), SOX9 (Sertoli), and CD34 (peritubular cells), and global miRNA expression was quantified using the NanoString nCounter Human v3b miRNA panel (827 miRNAs). Exploratory Ingenuity Pathway Analysis of miRNA data was used to identify predicted mRNA associations with a testis/reproductive disease filter. Results: hTTEs exhibited significantly greater IC_50_ values than matched 2D cultures for all three metals (*p* < 0.05), demonstrating greater resistance to acute heavy-metal cytotoxicity in the hTTE culture system, consistent with enhanced antioxidant buffering in the 3D microenvironment. Metal-associated changes in cell-type marker transcripts were distinct: Hg^2+^ produced broad reductions in markers associated with all four populations; Pb^2+^ produced greater reductions in Sertoli and peritubular cell markers while relatively preserving spermatogonial and Leydig cell markers; and Tl^+^ reduced somatic support cell markers with relative enrichment of spermatogonial (~7-fold) and Leydig cell marker expression. Heavy-metal exposure was associated with exploratory miRNA changes previously linked to redox imbalance, BCL2-mediated apoptosis, MYC/CDKN1A-driven nucleolar stress, AHR/CYP1A1 activation, and EGFR/COX-2 signaling. Conclusions: These findings demonstrate greater resistance of the hTTE culture system to acute heavy-metal cytotoxicity and suggest metal-specific differences in testicular cell-type responses. Within the established redox biology of Hg^2+^, Tl^+^, and Pb^2+^, the observed patterns provide a framework for investigating oxidative stress mechanisms directly in a human-relevant testicular model. This system may provide a platform for future studies incorporating direct redox measurements and antioxidant rescue strategies.

## 1. Introduction

Male factor abnormalities account for approximately half of all infertility cases worldwide, and a growing body of evidence points to oxidative stress (OS) as a unifying, mechanistically tractable pathway through which diverse environmental and occupational exposures impair male fertility [1]. Within the testis, physiological levels of reactive oxygen species (ROS) and reactive nitrogen species (RNS) are required for steroidogenesis, sperm capacitation, hyperactivation, and the acrosome reaction; when antioxidant defenses are overwhelmed, however, the same chemistry damages lipids, proteins, and sperm DNA; disrupts the blood–testis barrier; and impairs spermatogenesis [1,2,3]. Heavy-metal pollutants—lead (Pb^2+^), mercury (Hg^2+^), and thallium (Tl^+^) among them—are well-established generators of testicular OS and have become subjects of intensive study in male reproductive toxicology.

Heavy metals impair the testis through several interrelated redox mechanisms. Redox-active metals such as Fe and Cu generate hydroxyl radicals through Fenton/Haber–Weiss chemistry, while thiol-reactive metals such as Hg^2+^, Pb^2+^, As, and Cd deplete glutathione (GSH), inhibit selenoenzymes (glutathione peroxidase, thioredoxin reductase), and bind sulfhydryl groups on antioxidant enzymes, mitochondrial respiratory complexes, and spermatogenic membrane proteins [4,5,6]. The resulting mitochondrial dysfunction promotes NADPH oxidase (NOX) activation, lipid peroxidation of polyunsaturated fatty acid-rich spermatogenic membranes, 8-hydroxy-2′-deoxyguanosine (8-OHdG) formation in sperm DNA, and the induction of Nrf2/Keap1-dependent transcriptional programs that ultimately fail to compensate for chronic insult [2,3,6]. Within the seminiferous epithelium, Sertoli, Leydig, peritubular, and germ cells each express a distinct complement of antioxidant defenses (SOD1/2, catalase, glutathione peroxidases, peroxiredoxins, thioredoxin, Nrf2), and accumulating evidence from rodent and human studies suggests that this heterogeneity translates into cell-type–specific vulnerability to metal-induced OS [4,7].

Lead is the most abundant heavy metal of the Earth’s crust and has been extensively mobilized into the environment by industrial activity and the demand for fossil fuels. Chronic Pb^2+^ exposure disrupts the hypothalamic–pituitary–testicular axis and impairs sperm function in both humans and rodents [1]. In mice, Pb^2+^ exposure at low doses elicits gross histopathologic changes within the testis, abnormal sperm morphology, and dysregulation of Sertoli cell gene programs [8,9]; mechanistically, these effects have been linked to GSH depletion, inhibition of antioxidant enzymes, zinc displacement from sulfhydryl-rich proteins, and lipid peroxidation [10,11]. Similarly, Hg^2+^ (particularly Hg^2+^ and methylmercury) accumulates in Sertoli and Leydig cells, depletes GSH and selenoenzyme activity, and induces mitochondrial ROS that drive apoptosis of germ and somatic testicular cells [4,7]. Tl^+^ exerts mitochondrial-targeted toxicity—uncoupling oxidative phosphorylation, competing with K^+^ for transport, and inducing nucleolar stress and BCL2-/MYC-/CDKN1A-mediated apoptotic programs—yet its effects on human testicular tissue remain poorly defined [12,13,14,15,16].

Because of the toxicity of even low-dose heavy-metal exposure in humans, direct in vivo mechanistic studies are not feasible, and clinical observational studies—although informative—cannot easily isolate metal-specific, dose–response, or cell-type–specific contributions to infertility. Most mechanistic work has therefore relied on rodent models and 2D human testicular cell cultures, both of which only partially recapitulate the cellular interactions and architectural compartmentalization of the seminiferous epithelium. To address this gap, our group previously developed a three-dimensional human testicular organoid model, termed the Human Testis Tissue Equivalent (hTTE), constructed from passaged adult human testicular cells reseeded onto solubilized human testicular extracellular matrix [17,18,19]. The hTTE supports testosterone production, partial spermatogenesis, and greater resistance to common gonadotoxic chemotherapeutics than matched 2D cultures, supporting its potential utility as a human-relevant platform for reproductive toxicology and therapeutic screening.

In the present study, we leveraged the hTTE platform to evaluate heavy-metal cytotoxicity and cell-type–associated responses to Hg^2+^, Tl^+^, and Pb^2+^ within the established oxidative stress biology of these toxicants. We compared concentration–response viability between hTTEs and matched 2D cultures, quantified changes in cell-type–associated marker transcripts by digital PCR, and profiled global miRNA responses to explore exposure-associated regulatory patterns linked to oxidative stress, apoptosis, DNA damage, and cell-cycle pathways. Together, these complementary endpoints were used to evaluate the hTTE as a human-relevant experimental system for resolving metal-specific toxicologic responses across multiple levels of analysis. These findings extend the established oxidative stress biology of Hg^2+^, Tl^+^, and Pb^2+^ into a human testicular model and provide a foundation for the mechanistic investigation of redox-associated reproductive injury. We emphasize that the present study directly assessed viability, cell-type–associated transcript abundance, and miRNA associations; oxidative stress is invoked here as an interpretive framework grounded in the established redox chemistry of these metals rather than as a directly measured endpoint in this model.

## 2. Materials and Methods

### 2.1. Human Materials

Whole testes from three adult brain-dead organ donors were obtained through the National Disease Research Interchange (NDRI). Relevant donor characteristics are provided in Supplementary Table S1. None of the donors had a documented history of testicular pathology, malignancy, chemotherapy, or radiation exposure, and the testicular tissue was classified as normal during NDRI procurement. Donors ranged from 18 to 56 years of age. Experiments were performed independently using tissue-derived cells from each of the three donors, with each donor treated as a biological replicate.

Upon receipt, testes were divided into small fragments (2–5 mm). These were cryopreserved in 1× minimal essential medium containing 20% fetal bovine serum and 8% dimethyl sulfoxide using a slow-freezing Mr. Frosty container (Thermo Fisher Scientific, Waltham, MA, USA) in a −80 °C freezer overnight. Following freezing, the tissue samples were stored long-term in liquid nitrogen. Testis decellularization and extracellular matrix extraction were performed as reported previously [18].

### 2.2. Testis Cell Isolation and Culture

**Figure 1:**
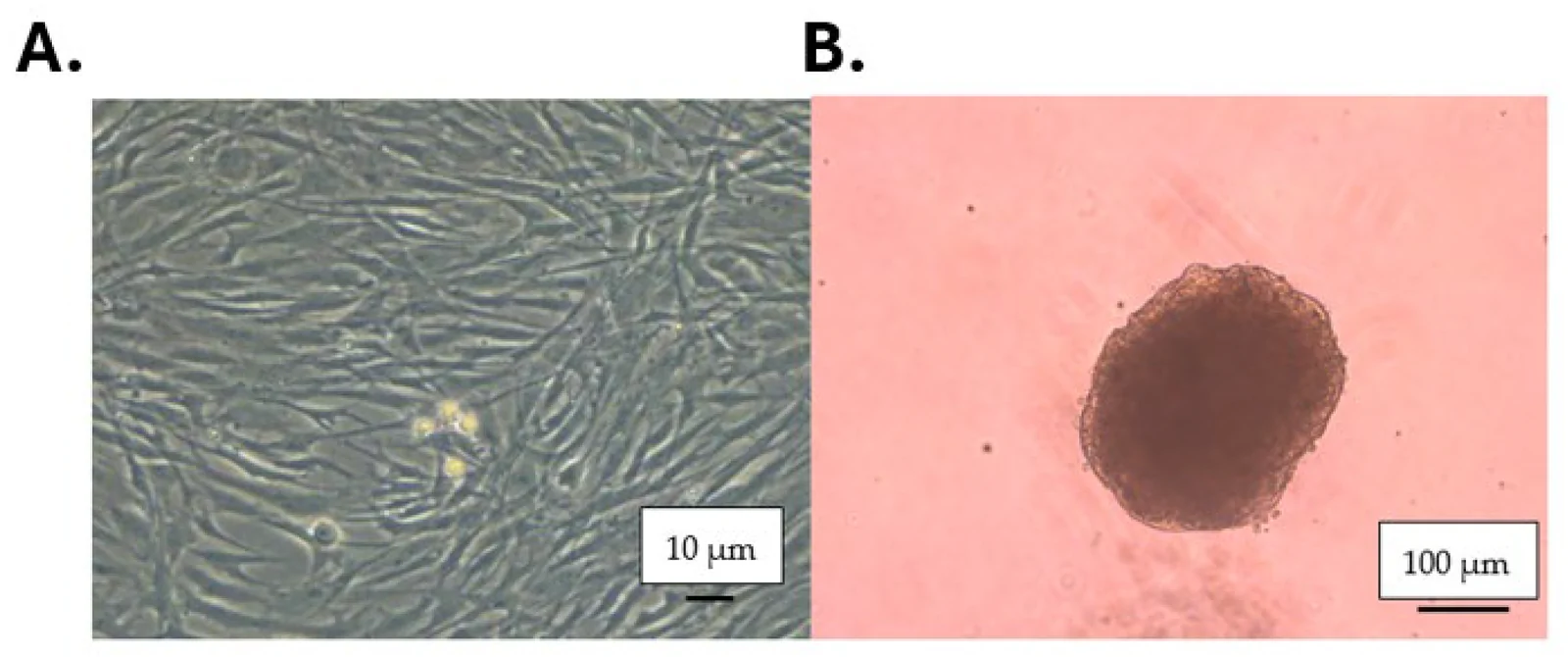
Images from testicular cell culture and hTTE assembly. (**A**) Spermatogonial stem cells are resting on a feeder-like layer of fibroblast-like testicular stromal cells before passage. (**B**) hTTEs after 48 h of self-assembly in low-attachment U-bottom plates; cells have compacted into spheroidal three-dimensional structures.

Testis tissue was removed from cryostorage, thawed, and digested enzymatically with trypsin, DNase, and collagenase; then, it was cultured for 4–5 weeks, as described previously (Figure 1) [17,19]. At passage 5 or 6, cells were split for hTTE and 2D culture comparisons. A portion of the cells harvested was briefly centrifuged to form a pellet, which was then flash-frozen for later RNA isolation and cell characterization.

Cells to be used for the 2D culture comparison were plated at 10,000 cells/well in 48-well plates (Falcon) in 250 µL of StemPro (Thermo Fisher Scientific, Waltham, MA, USA) Complete medium and incubated for 48 h at 37 °C, 5% CO_2_.

### 2.3. Testis Cell Characterization via PCR

RNA was isolated from flash-frozen cell pellets collected previously using the miRNeasy RNA extraction kit (Qiagen, Germantown, MD, USA). RNA was additionally isolated from flash-frozen whole testis tissue (WT) corresponding to each of the original cell cultures, following homogenization using a mortar and pestle and a Qiashredder (Qiagen). The RNA concentration and quality were evaluated using a Nanodrop Fluorometer. RNA isolated from whole testis tissue and 2D culture was converted to cDNA using a high-capacity cDNA reverse transcription kit (Life Technologies, Carlsbad, CA, USA). Characterization was performed via real-time quantitative PCR (rt-qPCR) using the following ABI TaqMan primers: ZBTB16 (Hs00232313_m1), UCHL1 (Hs00985157_m1), and THY1 (Hs00174816_m1) for undifferentiated spermatogonia; GATA4 (Hs00171403_m1), SOX9 (Hs00165814_m1), and CLU (Hs00971656_m1) for Sertoli cells; STAR (Hs00264912_m1), TSPO (Hs00559362_m1), and CYP11A1 (Hs00897320_m1) for Leydig cells; CD34 (Hs02576480_m1) for peritubular cells; and POLR2A (Hs00172187_m1) as an endogenous control. For each assay, 50 ng of cDNA was used. The cycling conditions were as follows: 95 °C for 10 min, 95 °C for 15 s (40 cycles), and 60 °C for 1 min. All runs were performed in duplicate. The expression of all genes was normalized to POLR2A, and relative expression was determined using the ΔΔCT method. PCR products were additionally evaluated by gel electrophoresis.

### 2.4. Formation of Three-Dimensional HTTEs

Cells to be used in hTTE formation were centrifuged at 350× *g* with the brake for 5 min and then re-suspended to a concentration of 1 × 10^6^ cells/mL in hTTE formation media: supplemented Stem Pro 34 (Invitrogen, Carlsbad, CA, USA) containing 30% FBS, 0.5% penicillin–streptomycin (Invitrogen), 1 µg/mL solubilized human testis ECM, 50 µg/mL gentamicin (ThermoFisher), 20 ng/mL recombinant human epidermal growth factor (Sigma-Aldrich, St. Louis, MO, USA), 10 ng/mL recombinant human glial cell line-derived neurotrophic factor (Sigma-Aldrich), and 10 ng/mL recombinant human leukemia inhibitory factor (LIF). Next, 100 µL/well of this cell suspension was seeded into all wells of a 96-well Ultra Low Attachment U-Bottom culture plate (Thermo Fisher), and the plates were centrifuged at 300× *g* for 1 min with the brake (Figure 1B). Plates were then moved to humidified incubators at 37 °C containing 5% CO_2_ and cultured for 48 h. The ECM batch and passage number of cells were controlled prior to hTTE formation.

### 2.5. Heavy-Metal Exposure and IC_50_ Determination

To compare the heavy-metal cytotoxicity between culture systems, concentration–response relationships and IC_50_ values were determined independently for hTTE and matched 2D cultures following a 48 h exposure to heavy-metal–supplemented media. Stock solutions of 150 mM, 300 mM, and 28.2 mM in ultrapure water (Invitrogen) were made for mercury chloride (HgCl_2_), thallium nitrate (TlNO_3_), and lead chloride (PbCl_2_), respectively. The StemPro media formation medium and Complete StemPro medium were mixed with heavy-metal stock solutions to generate increasing micromolar concentrations of heavy-metal–supplemented media. For each metal, cultures were exposed across the graded concentration series shown in Table 1. Viability was normalized to donor-matched vehicle controls (0–100%), and IC_50_ values were estimated separately for each donor by fitting a four-parameter, variable-slope log (inhibitor) versus response model in GraphPad Prism 8 (mean R^2^ > 0.9). The donor-matched 2D and hTTE cultures (identical donor and passage number) were compared, and the three per-donor IC_50_ estimates per metal were used for the 2D-vs-hTTE statistical comparison. Comparisons were made using a paired *t*-test for each metal, consistent with the paired-sample approach used for digital PCR (Section 2.8). Medium was added to existing culture wells to a total medium volume of 200 µL for hTTEs (adding 100 µL of 2× condition concentration) and 250 µL for 2D culture (complete medium refreshment). Exposures for each concentration were performed in quadruplicate for hTTE and 2D cultures. All conditions were incubated for 48 h at 37 °C.

Following incubation, cell viability was assessed using the CellTiter-Glo Luminescent Cell Viability Assay (Promega, Fitchburg, WI, USA) as an ATP-based measure of viable cells. To perform this, all but 100 µL of medium was removed from the target wells, and 100 µL of ATP reagent was added. Plates were gently shaken for 5 min and then incubated, covered, at room temperature for 25 min. The entire contents of the wells to be tested were transferred to a luminescence assay-compatible 96-well plate (Nalgene, Rochester, NY, USA). The signal was measured using a Veritas Luminometer (Promega). IC_50_ values were then determined from these data using Prism 8 (GraphPad, San Diego, CA, USA).

Following initial IC_50_ determination, hTTEs were exposed for 48 h to selected concentrations for downstream characterization. For Tl^+^ and Pb^2+^, the fitted hTTE IC_50_ concentrations were used. For Hg^2+^, the initial concentration–response analysis yielded a fitted hTTE IC_50_ of 276.9 µM. When this concentration was subsequently applied in a downstream exposure, Live/Dead assessment demonstrated near-complete cell death rather than the anticipated intermediate viability. The Hg^2+^ concentration was therefore reduced to 225 µM for subsequent downstream analyses. Accordingly, the 225 µM Hg^2+^ condition represents an experimentally adjusted downstream exposure concentration rather than a separately fitted IC_50_. Following exposure, hTTEs were harvested for miRNA isolation using the miRNeasy RNA extraction kit (Qiagen). The RNA concentration and quality were evaluated using the Nanodrop Fluorometer.

### 2.6. Live/Dead Imaging

Live/Dead imaging was performed on hTTEs exposed to the selected downstream heavy-metal concentrations described above, as well as on controls, using the Molecular Probes Live-Dead Cell Imaging kit (Thermo Fisher). hTTEs were harvested along with their media, and 2 µL of calcein dye (1:80 in DMSO) and 4 µL of ethidium homodimer-1 dye were added. The samples were then briefly agitated to mix and incubated at 37 °C for 30 min. hTTEs were then imaged on an Olympus FV10i macro-confocal microscope. Live/Dead imaging was used as a qualitative assessment of viability and spatial cellular injury.

### 2.7. miRNA Analysis

To assess changes in miRNA expression following heavy-metal exposure, miRNA analysis was performed using the Nanostring nCounter Human v3b miRNA panel (NanoString, North, Seattle, WA, USA), with probes representing 827 unique miRNAs. For each donor, samples consisted of a control, Hg^2+^ (225 µM), Tl^+^ (fitted hTTE IC_50_), and Pb^2+^ (fitted hTTE IC_50_), yielding 3 biological replicates per condition and 12 samples total. A total of 100 ng of RNA was used per sample. Per the manufacturer’s instructions, specific DNA miR tags were ligated to the 3′ ends of sample miRNAs. The samples were hybridized after excess tags were removed using miR:tag-specific capture and reporter probes. Hybridization was carried out at 64 °C for 18 h, and the hybridized samples were loaded onto a streptavidin-coated nCounter Sprint cartridge. The cartridge was then read on the nCounter Sprint Profiler (NanoString, North, Seattle, WA, USA), which counted individual fluorescent barcodes attached to miRNA targets.

The miRNA target normalization was carried out with the geometric mean of the top 100 miRNAs detected and with background subtraction of 57 counts (the average of the exogenous negative controls in the panel plus two standard deviations). Following normalization, all miRNAs with average counts of <5 within a sample group were excluded to ensure robust expression within the sample. The miRNA analysis results were normalized using nSolver 4.0 (NanoString) and analyzed within nSolver.

Geometric mean normalization to the top 100 detected miRNAs was used because panel housekeeping controls did not demonstrate sufficient cross-sample stability for normalization in this dataset. Differential expression was evaluated using an unadjusted *p* < 0.05 threshold, with multiple-testing correction performed using the Benjamini–Hochberg false discovery rate (FDR) method. No differentially expressed miRNAs remained statistically significant following FDR correction.

Qiagen Ingenuity Pathway Analysis (IPA; Spring Release, 25 April 2026) was therefore used as an exploratory, hypothesis-generating analysis of nominal expression patterns. IPA associations represent database-predicted miRNA–mRNA relationships rather than experimentally measured mRNA changes in this study. Only miRNAs meeting prespecified nominal differential expression criteria between control and exposed hTTEs were included in the exploratory analysis. A miRNA target filter was used to select for miRNA–mRNA relationships that were associated with reproductive disease, localized to testis cells, and experimentally observed. These mRNAs were then cross-referenced in the literature for any association with exposure to the tested heavy metals.

### 2.8. Digital PCR

Digital PCR was performed on samples used for miRNA analysis to quantify changes in the relative abundance of testicular cell-type–associated transcripts following heavy-metal exposure. Digital PCR was performed using the QuantStudio 3D Digital PCR System (Thermo Fisher Scientific, Waltham, MA, USA), with 25 ng of cDNA per chip (Catalog #A26316). Chips were loaded with 7.3 µL of 3D Digital PCR Master Mix, 0.8 µL each of target FAM and POLR2A VIC (Hs00172187_m1) TaqMan primers, and up to 5.6 uL of sample plus ultra-pure distilled water. The target primers used were ZBTB16 (Hs00232313_m1) for undifferentiated spermatogonia, STAR (Hs00264912_m1) for Leydig cells, SOX9 (Hs00165814_m1) for Sertoli cells, and CD34 (Hs02576480_m1) for peritubular cells. The cycling conditions were as follows: 95 °C for 10 min, 95 °C for 15 s (40 cycles), and 60 °C for 1 min. Chips were brought to room temperature following removal from the thermocycler and were then read by the QuantStudio 3D Chip Reader. Following uploading to the ThermoFisher Cloud (https://www.thermofisher.com/us/en/home/digital-science.html, accessed on 10 November 2025), the chips were evaluated using the dPCR Analysis Suite. Target transcript abundance was normalized to POLR2A (VIC) and expressed relative to the donor-matched vehicle control. Because exposed and control samples were donor-matched, statistical comparisons were performed using paired *t*-tests.

## 3. Results

### 3.1. Testis Cell Characterization

**Figure 2:**
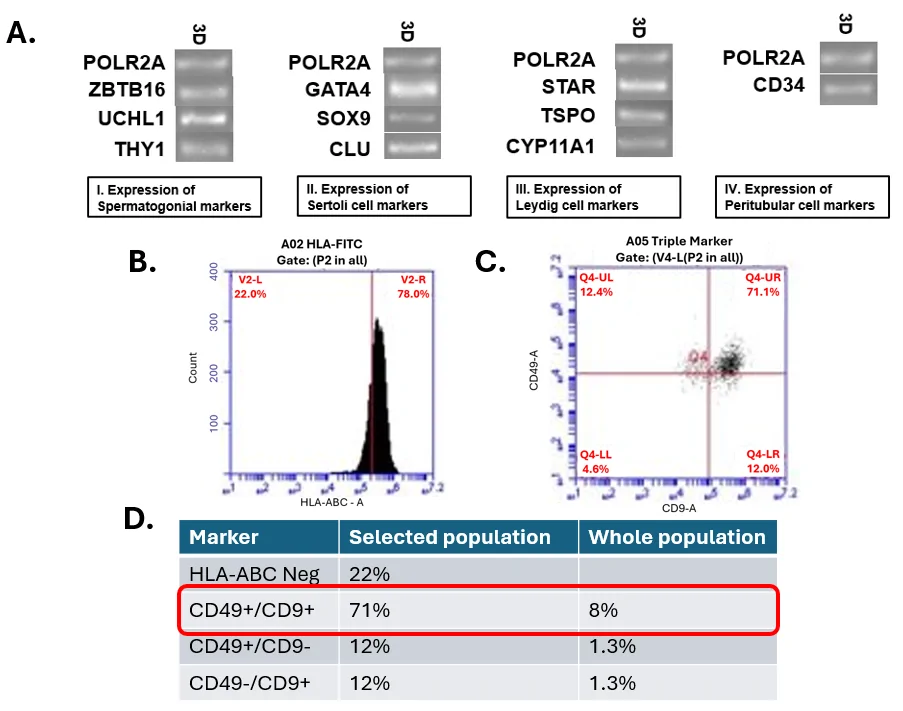
Characterization of the testicular cells incorporated into hTTEs. (**A**) Gel electrophoresis of RT-qPCR products across the three donor cell lines, using POLR2A as the endogenous control and ZBTB16, UCHL1, and THY1 as markers of spermatogonia; GATA4, SOX9, and CLU as Sertoli cell markers; STAR, TSPO, and CYP11A1 as Leydig cell markers; and CD34 as a peritubular cell marker. (**B**–**D**) Flow cytometry confirming the presence of CD49^+^/CD9^+^ spermatogonia (red box) in 2D culture prior to incorporation into hTTEs, comprising approximately 8% of the cell population in the representative sample shown.

RT-qPCR verified the presence of transcripts associated with spermatogonia, Leydig cells, Sertoli cells, and peritubular cells in the hTTEs (Figure 2A). Flow cytometry demonstrated the presence of spermatogonia in the 2D cultures prior to incorporation into hTTE formation (Figure 2B–D).

### 3.2. The hTTE Culture System Exhibits Greater Resistance to Heavy-Metal Cytotoxicity

**Figure 3:**
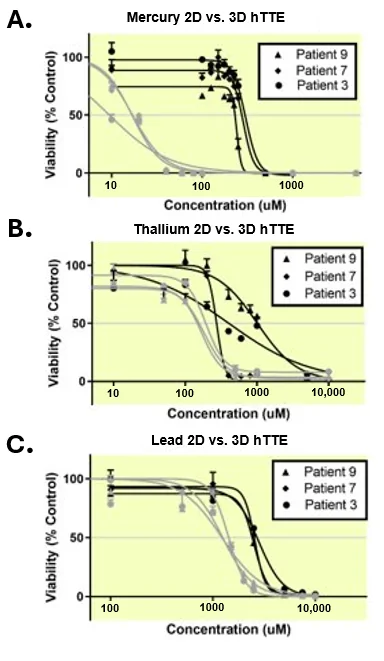
Comparison of IC_50_ values between 2D (gray lines) and hTTE (black lines) culture systems for (**A**) mercury, (**B**) thallium, and (**C**) lead. In each case, the hTTE culture system exhibited a higher mean IC_50_ across all three donors than the matched 2D culture.

For each of the three heavy metals tested, hTTEs exhibited significantly greater resistance to acute cytotoxicity than matched 2D cultures, as indexed by the IC_50_ values from ATP-based viability assays (*p* < 0.05). The calculated IC_50_ values are reported in Table 1 and shown graphically in Figure 3A–C; the hTTE system exhibited approximately 2–5-fold higher IC_50_ values than the matched 2D cultures across donors.

### 3.3. Live/Dead Imaging Reveals a Diffuse, Non-Eccentric Pattern of Cell Death

**Figure 4:**
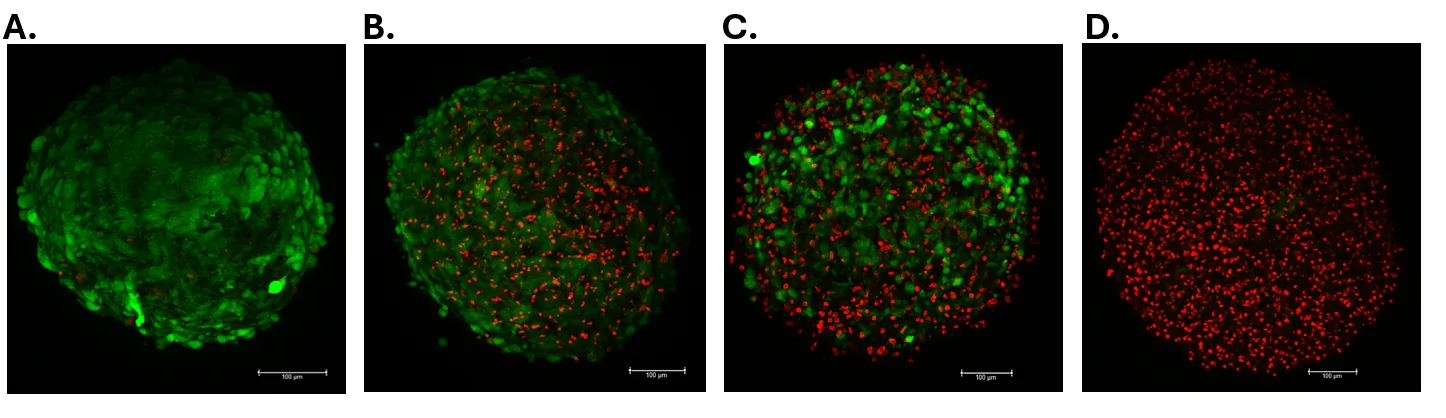
Confocal Live/Dead viability imaging of hTTEs after 48 h of heavy-metal exposure. Live/Dead images are qualitative and representative of three biological replicates; no fluorescence intensity or cell-count quantification was performed. (**A**) Vehicle-treated hTTE control; (**B**) hTTE exposed to the fitted hTTE IC_50_ concentration of thallium nitrate; (**C**) hTTE exposed to the fitted hTTE IC_50_ concentration of lead chloride; (**D**) hTTE exposed to 225 µM mercury chloride. Live cells are labeled with calcein-AM (green), and compromised cells are labeled with ethidium homodimer-1 (red). Scale bars = 100 µm in all panels.

Following exposure to the selected downstream heavy-metal concentrations, Live/Dead confocal imaging demonstrated the presence of both viable and nonviable cells throughout the hTTE constructs, with heavy-metal–associated cytotoxicity distributed across the constructs rather than confined to a peripheral or central region (Figure 4A–D).

### 3.4. Heavy-Metal Exposure Is Associated with Distinct miRNA Profiles in hTTEs

Following background subtraction and normalization, 110, 119, 116, and 128 miRNAs were detected in control, mercury-, thallium-, and lead-exposed hTTEs, respectively. Comparative analysis identified 7, 7, and 8 miRNAs with nominally significant exposure-associated differences (*p* < 0.05) following mercury, thallium, and lead exposure, respectively. Although these associations did not remain significant following FDR correction (all adjusted *p* > 0.05), the nominally significant miRNAs were carried forward as an exploratory, hypothesis-generating dataset for the evaluation of exposure-associated biological pathways. Two complementary comparisons were performed relative to control: a detection-level (presence/absence) analysis of miRNAs gained or lost with exposure (Table 2A), and a differential expression analysis of miRNAs commonly detected in both the control and exposed groups (Table 2B).

At the detection level, exposure was associated with a gain of 12, 9, and 20 miRNAs and the loss of 3, 3, and 2 miRNAs relative to the control for mercury, thallium, and lead, respectively (Table 2A—Panel A). Rather than being metal-restricted, most of these species were shared across conditions: six miRNAs (miR-106b-5p, miR-1290, miR-181b-5p/miR-181d-5p, miR-299-3p, miR-320e, and miR-494-3p) were gained in all three exposure groups, and one (miR-181a-3p) was lost in all three, suggesting a shared exposure-associated detection pattern. Only a minority were restricted to a single metal: miR-30a-3p (mercury), miR-1973 (thallium), and nine species detected only after lead exposure, with lead accounting for the largest number of uniquely gained miRNAs. Because this analysis reflects the presence or absence relative to the defined detection threshold, these findings describe exposure-associated detection patterns rather than changes in expression magnitude.

Among the miRNAs commonly detected in the control and exposed groups, 7, 7, and 8 were nominally differentially expressed (*p* < 0.05) after mercury (5 down, 2 up), thallium (1 down, 6 up), and lead (4 down, 4 up) exposure, respectively (Table 2B). Two species were shared across conditions: miR-29b-3p was upregulated after both thallium and lead exposure, and miR-630 was upregulated after both mercury and lead exposure. Nominally significant miRNAs and their direction of change are summarized in Table 2B; none remained significant following FDR correction.

The nominally differentially expressed miRNAs were further evaluated using Ingenuity Pathway Analysis (IPA) to identify candidate downstream mRNA relationships associated with the exposure-specific miRNA profiles. IPA identified 17, 146, and 192 candidate mRNA associations for mercury, thallium, and lead, respectively, of which 2, 6, and 14 had been previously reported in the heavy-metal literature (Table 3). These predicted relationships provide candidate molecular targets for further investigation of metal-specific responses in the hTTE.

### 3.5. Digital PCR Identifies Metal-Specific Changes in Cell-Type–Associated Transcripts

**Figure 5:**
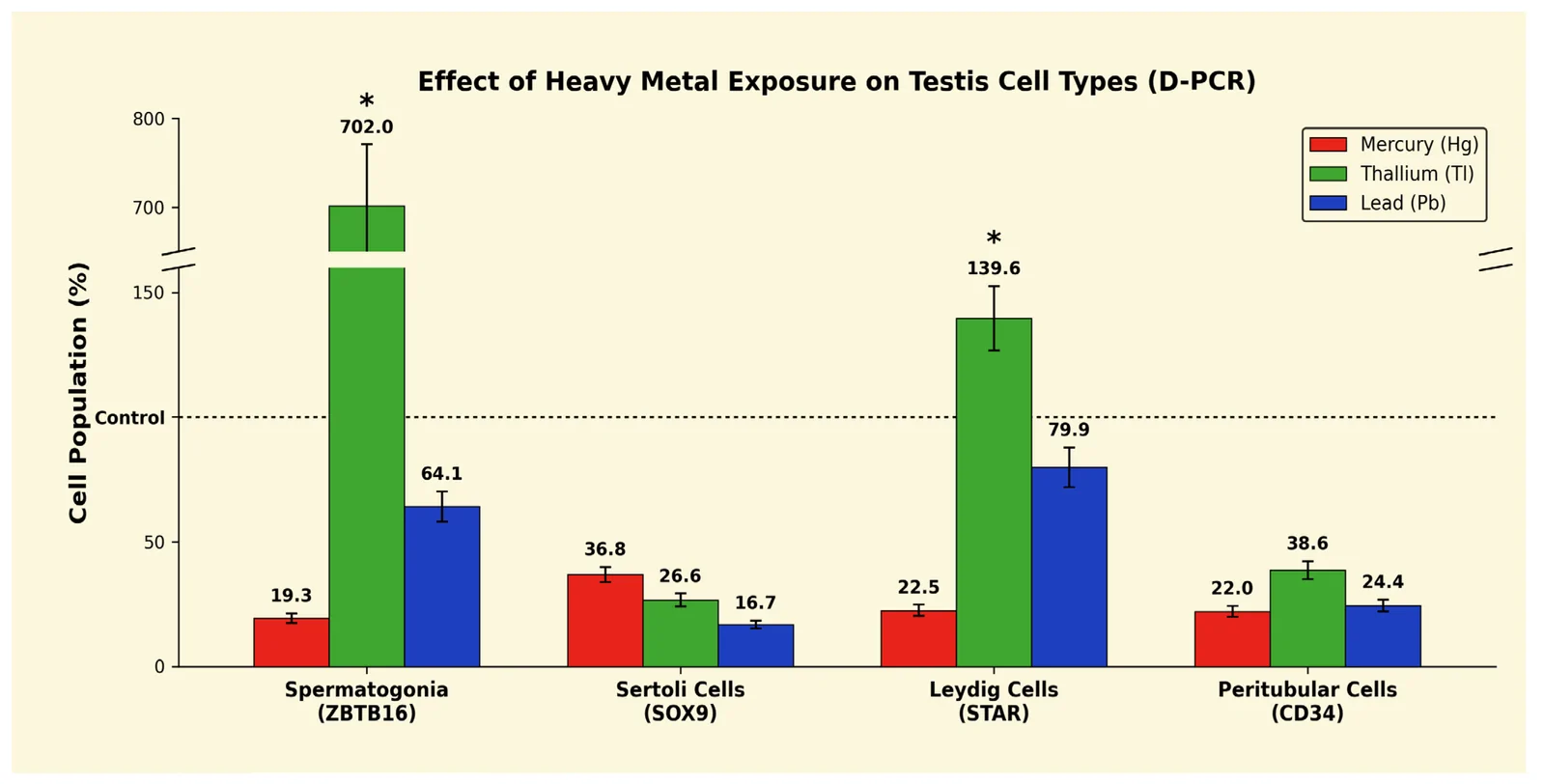
Digital PCR analysis of cell-type–associated marker transcripts following heavy-metal exposure. Expression is shown relative to donor-matched vehicle controls. Thallium exposure was associated with marked relative enrichment of the spermatogonial-associated marker ZBTB16 and a smaller relative enrichment of the Leydig cell-associated marker STAR. Error bars represent the SEM (*n* = 3 donors); * *p* < 0.05 by paired *t*-test versus donor-matched vehicle control.

Digital PCR for cell-type–associated marker transcripts indicated distinct exposure-associated patterns within hTTEs (Figure 5). Mercury (Hg^2+^) exposure was associated with reductions in all four marker transcripts: ZBTB16, STAR, and CD34 decreased to approximately 20% of vehicle control levels, while SOX9 decreased to approximately 37% of control. Lead (Pb^2+^) exposure produced a different pattern, with SOX9 and CD34 decreasing to approximately 40% and 50% of control, respectively, while ZBTB16 and STAR were relatively preserved at approximately 64% and 80% of control. Thallium (Tl^+^) exposure generated the most distinctive pattern, with ZBTB16 increasing to approximately 700% of the donor-matched control level and STAR also showing relative enrichment, while SOX9 and CD34 were reduced. These changes represent the relative abundance of cell-type–associated transcripts and should not be interpreted as direct measurements of cell number or proliferation. Comparisons between exposed and donor-matched vehicle control conditions were evaluated by paired *t*-test (Section 2.8); only the thallium-associated increases in ZBTB16 and STAR reached statistical significance (*p* < 0.05).

## 4. Discussion

Heavy-metal exposure is a leading modifiable contributor to oxidative stress-driven male infertility, yet most mechanistic insights derive from rodent models or 2D human cultures that fail to recapitulate the cellular interactions and architectural organization of the seminiferous epithelium. In this study, we used a three-dimensional Human Testis Tissue Equivalent (hTTE) to compare the responses of human testicular tissue to Hg^2+^, Tl^+^, and Pb^2+^ with those of matched 2D cultures. Across three human donor-derived preparations, the hTTE culture system demonstrated greater resistance to acute heavy-metal cytotoxicity, while digital PCR and exploratory miRNA profiling identified distinct exposure-associated response patterns. Together, these findings extend established heavy-metal reproductive toxicology into a human 3D testicular model and support the hTTE as a platform for investigating the mechanisms of testicular injury and potential protective interventions.

When hTTEs and matched 2D cultures were challenged side-by-side, the IC_50_ values for all three metals were 2- to 5-fold higher in hTTEs than in 2D cultures (Figure 3; Table 1), recapitulating our prior observation with gonadotoxic chemotherapeutics [18]. The consistency of this effect across three chemically distinct toxicants suggests that features of the hTTE culture system confer greater resistance to acute cytotoxic injury. Its three-dimensional organization, multicellular interactions, extracellular matrix, and culture microenvironment may contribute to this response. Live/Dead imaging demonstrated cell death throughout the hTTE volume rather than exclusively at its periphery (Figure 4), supporting the penetration of injury throughout the construct rather than an exclusively surface-limited effect. Because the hTTE and 2D culture systems also differ in medium composition, including ECM and growth factor supplementation, the relative contributions of the architecture and culture composition cannot be separated in the present study.

The metal-specific, cell-type–associated transcript patterns can be interpreted in the context of established reproductive toxicology. Lead induces testicular oxidative stress through GSH depletion, antioxidant enzyme inhibition, and displacement of essential metals such as zinc [10,11], while rodent studies demonstrate injury to the seminiferous epithelium and Sertoli cells [11]. This is directionally consistent with the reduction in Sertoli and peritubular-associated transcripts observed following Pb^2+^ exposure. Hg^2+^ similarly depletes GSH and selenoenzymes and induces mitochondrial ROS and lipid peroxidation [4], with deposition reported in both Sertoli and Leydig cells [7]. Correspondingly, Hg^2+^ exposure produced broad reductions across all four cell-type marker transcripts in the hTTE, suggesting a comparatively widespread pattern of testicular injury.

Thallium toxicity to the testis is comparatively underexplored. Early rat studies reported germ cell loss and Sertoli cell abnormalities following Tl^+^ exposure [12]. In contrast, hTTEs demonstrated an approximately seven-fold relative increase in the spermatogonial-associated marker ZBTB16, together with reductions in Sertoli and peritubular-associated transcripts (Figure 5). This should not be interpreted as stimulation of spermatogonial proliferation; it may reflect relative enrichment following changes in other cellular compartments or altered marker expression per cell. Tl^+^ is known to disrupt mitochondrial K^+^ homeostasis and oxidative phosphorylation and to activate apoptotic and cell-cycle pathways [13,14,15,16]. The distinct response observed here identifies Tl^+^ as particularly interesting for subsequent cell-resolved investigation in the hTTE and highlights the ability of the model to distinguish exposure-specific patterns that may be obscured by whole-tissue viability measurements alone.

Collectively, the ability of the hTTE to generate quantitative concentration–response relationships while supporting imaging, cell-type–associated molecular analysis, and miRNA profiling demonstrates the versatility of this human testicular model for reproductive toxicology. An important next step will be to determine whether the exposure-associated patterns observed here correspond to directly measurable changes in redox state and whether they can be modified pharmacologically. Prior studies have reported protective effects of antioxidant strategies against heavy-metal–associated reproductive injury [13,14,15,16]. The exploratory miRNA analysis also identified candidate regulatory relationships relevant to established heavy-metal biology. Hg^2+^-associated miRNAs identified a candidate relationship involving PTEN signaling, in line with reports of altered PTEN regulation in mercury-exposed glial tissue [20]. Lead-associated miRNAs were associated with a broader predicted network that included redox-sensitive transcriptional programs (AHR/CYP1A1 activation [21,22,23,24]), EGFR/COX-2 inflammatory and lipid peroxidation pathways [25,26,27,28,29], lipid metabolism shifts (SCD downregulation [25]), epigenetic remodeling (CDKN2A methylation, DNMT3A modulation [30,31,32,33]), BCL2-mediated apoptosis [32,33,34], and dysregulation of SIRT1/mTOR-controlled autophagy and Wnt/β-catenin signaling [20,35]. These associations provide biologically plausible candidates for subsequent mechanistic investigation of redox-sensitive signaling in the hTTE.

***Therapeutic implications:*** The distinct heavy-metal responses observed in the hTTE, together with the established role of oxidative stress in Hg^2+^-, Tl^+^-, and Pb^2+^-associated toxicity, generate a directly testable hypothesis that pharmacological augmentation of testicular antioxidant defenses may modify these injury patterns. Candidate agents for future hTTE rescue screening include N-acetylcysteine and γ-glutamylcysteine ethyl ester (GSH precursors), selenium and selenomethionine (selenoenzyme cofactors), α-tocopherol, ascorbate, melatonin, coenzyme Q10, and Nrf2/Keap1 axis activators (sulforaphane, dimethyl fumarate). Rodent and human seminal studies have already shown partial reversal of Pb^2+^- and Hg^2+^-induced spermatogenic damage with vitamin C, vitamin E, and zinc supplementation [10,11], aligning with the broader clinical hypothesis—central to this Special Issue [36]—that antioxidant therapy may mitigate oxidative stress–associated male reproductive injury. The hTTE provides a physiologically relevant human testicular platform in which these hypotheses can be tested across defined exposure and intervention dose–response relationships while integrating cellular, molecular, and functional endpoints.

***Limitations and future directions:*** This study has several limitations that also define clear next steps for investigation. First, our cell-type readout was restricted to a panel of marker transcripts (ZBTB16, STAR, SOX9, CD34). Digital PCR quantifies lineage-associated transcripts rather than cells directly, and heavy-metal exposure could alter per-cell expression independently of cell number. The observed patterns should therefore be interpreted as changes in relative marker transcript abundance and can be further resolved using orthogonal single-cell or immunostaining-based approaches. Similarly, the miRNA analysis did not directly measure corresponding mRNA targets. Second, the present study evaluated heavy-metal cytotoxicity and associated molecular responses within the established oxidative stress biology of these toxicants but did not directly quantify redox state. Future incorporation of intracellular ROS (CM-H_2_DCFDA, MitoSOX), lipid peroxidation (MDA/TBARS or BODIPY-C11), GSH:GSSG ratio, 8-OHdG accumulation, mitochondrial membrane potential, Nrf2 nuclear translocation, or SOD/CAT/GPx activity will enable direct mechanistic interrogation of oxidative stress pathways in the hTTE and establish how these pathways relate to the exposure-specific cellular and molecular responses identified here. Third, our miRNA study included three biological replicates per condition, and no significant differential expression survived false-discovery correction; additional donor-derived experiments will be required to validate the exploratory patterns identified here and to assess inter-donor variability. Fourth, downstream functional endpoints of steroidogenic and spermatogenic function were not measured in the exposed hTTEs. Endocrine disruption may occur below cytotoxic thresholds, and functional steroidogenic and differentiation endpoints represent important extensions of the present work. Together with direct redox measurements and antioxidant rescue experiments, these studies will further define the mechanistic and translational utility of the hTTE for human reproductive toxicology.

## 5. Conclusions

We present a three-dimensional Human Testis Tissue Equivalent (hTTE) that exhibits significantly greater resistance than matched 2D cultures to acute cytotoxicity from mercury, thallium, and lead and demonstrates distinct heavy-metal–associated patterns in cell-type–associated marker transcripts across three human donors. Exploratory miRNA profiling identified exposure-associated patterns linked to redox, apoptosis, DNA damage, and cell-cycle pathways consistent with established heavy-metal biology. Taken together, these findings demonstrate the utility of the hTTE for resolving human testicular responses to environmental toxicants across multiple levels of analysis and support its continued development as a human-relevant platform for mechanistic reproductive toxicology and the evaluation of antioxidant and other protective interventions.

## Figures and Tables

**Table 1:** Calculated IC_50_ values for 2D and 3D culture systems for mercury, thallium, and lead.

Toxic Agent	2D Mean (SD)	3D Mean (SD)
Mercury	14.43 (4.31)	276.9 (28.5)
Thallium	181.33 (12.451)	378.15 (36.98)
Lead	1299.67 (110.726)	2638.33 (163.02)

**Table 2:** (**A**) miRNA detection summary (presence/absence relative to vehicle control). Panel A. Lists of the total number of miRNA counts detected for control and each condition, including number of miRNAs gained or lost compared to the control. Panel B. Classification of gained miRNA based on condition or combination of conditions. Panel C. Classification of lost miRNA based on condition or combination of conditions. (**B**) Differentially expressed miRNAs common to control and exposure (nominal *p* < 0.05).

(**A**)					
**Panel A—Detection counts per condition**					
**Condition**	**Total miRNAs detected**	**Gained vs. control (detected in exposure, absent in control)**		**Lost vs. control (detected in control, absent in exposure)**	
Control	110	—		—	
Mercury	119	12		3	
Thallium	116	9		3	
Lead	128	20		2	
**Panel B—Gained miRNAs classified by cross-metal sharing**					
**Sharing category**		**miRNA(s)**		***n***	
Gained in **all three** metals		miR-106b-5p, miR-1290, miR-181b-5p/miR-181d-5p, miR-299-3p, miR-320e, miR-494-3p		6	
Gained in Hg^2+^ + Pb^2+^		miR-126-3p, miR-151a-5p, miR-30b-5p, miR-642a-3p		4	
Gained in Hg^2+^ + Tl^+^		miR-4284		1	
Gained in Tl^+^ + Pb^2+^		miR-186-5p		1	
Gained in Hg^2+^ only		miR-30a-3p		1	
Gained in Tl^+^ only		miR-1973		1	
Gained in Pb^2+^ only		miR-1180-3p, miR-130b-3p, miR-146a-5p, miR-152-3p, miR-30d-5p, miR-30e-5p, miR-370-3p, miR-5196-3p/miR-6732-3p, miR-656-3p		9	
**Panel C—Lost miRNAs classified by cross-metal sharing**					
**Sharing category**		**miRNA(s)**		***n***	
Lost in **all three** metals		miR-181a-3p		1	
Lost in Tl^+^ + Pb^2+^		miR-154-5p		1	
Lost in Hg^2+^ only		miR-193a-5p/miR-193b-5p, miR-137		2	
Lost in Tl^+^ only		miR-485-3p		1	
(**B**)					
**Condition**	**miRNA**	**Direction**	**Condition**	**miRNA**	**Direction**
Mercury	miR-214-3p	↓	Thallium	miR-106a-5p/miR-17-5p	↑
Mercury	miR-1234-3p	↓	Thallium	miR-29b-3p	↑
Mercury	miR-4286	↓	Thallium	miR-26b-5p	↑
Mercury	miR-107	↓	Lead	miR-503-5p	↓
Mercury	miR-3195	↓	Lead	miR-143-3p	↓
Mercury	miR-630	↑	Lead	miR-125a-5p	↓
Mercury	miR-1246	↑	Lead	miR-34a-5p	↓
Thallium	miR-145-5p	↓	Lead	miR-4454/miR-7975	↑
Thallium	miR-574-3p	↑	Lead	miR-29b-3p	↑
Thallium	miR-374a-5p	↑	Lead	miR-630	↑
Thallium	miR-423-5p	↑	Lead	miR-148a-3p	↑

Significant increase (↑) or decrease (↓) in miRNA level.

**Table 3:** Summary of database-predicted mRNA associations identified by exploratory IPA analysis of miRNAs meeting nominal differential expression criteria (unadjusted *p* < 0.05) following heavy-metal exposure. mRNAs previously reported in association with corresponding heavy-metal exposure are underlined.

Heavy Metal	Associated mRNA Found
Mercury	AGO1, AGO2, AGO3, ATF4, BACE1, CCNE1, CDK6, CRKL, FBXW11, GPD1, ICOS, ING4, NFIA, POU4F2, PTEN, SCN3A, SERBP1
Thallium	ACBD3, ACVR2A, ADAMTS14, ADAMTS15, ADAMTS2, AHNAK, APP, ARID4B, BACE1, BAMBI, BCL2, BCL2L11, BLACAT1, BMPR2, BNIP2, CAMTA1, CAV2, CBFB, CCNA2, CCND1, CD276, CDC42, CDK4, CDK6, CDKN1A, CES1, CLINT1, CNOT8, COL15A1, COL1A1, COL1A2, COL3A1, COL4A1, COL4A2, COL4A5, COL5A1, COL5A2, COL5A3, COL8A1, CREB1, CRIM1, CRTAP, CXCL8, DDR1, DNMT3A, DNMT3B, DUSP2, E2F1, E2F2, E2F3, EGR2, EIF4E, EOMES, ESR1, F11R, FBN1, FLI1, FSCN1, GAS7, GMFB, GPR37, HBP1, HDAC4, HIPK3, IGF1R, IRS1, ITCH, JAK1, KLF4, KLF5, KRT7, LAMC1, LOXL2, LOXL4, MAPK7, MCL1, MDM2, MICA, MITF, MLF1, MMP3, MUC1, MYBL2, MYC, MYLIP, MYO5A, NASP, NAV3, NCOA3, NDUFA4, NID1, NID2, PADI1, PAK5, PARP8, PDGFA, PDGFB, PIK3R1, PKD2, PLEC, PPARG, PPM1D, PPP3CA, PTEN, PURA, RASA1, RB1, RBL2, RERE, ROBO4, RTKN, RUNX1, S1PR1, SHROOM2, SOX9, SP1, SPARC, SPTB, SRSF10, STAT3, SWAP70, TBC1D2, TBX21, TCL1A, TDG, TET1, TGFB3, TGFBR1, TGFBR2, TLR7, TNF, TNFAIP3, TP63, TPM3, TRIM9, TUBB2A, TYR, TYRP1, UNG, USP46, VCAN, VEGFA, VIM, YY1, ZBTB7A, ZFP36L1
Lead	ABTB1, ACSS1, ACVR2A, ADAMTS1, ADAMTS14, ADAMTS15, ADAMTS2, AGO1, AJUBA, ALOX5, ANLN, ARID3A, ARID3B, ATF6, AXIN2, BACE1, BAK1, BCL2, BMF, BMPR1A, BMPR1B, CASP6, CASP7, CAV2, CBFB, CBX7, CCKBR, CCN5, CCND1, CCND2, CCND3, CCNE1, CCNE2, CCR5, CD276, CD47, CDC14A, CDC25A, CDC42, CDH5, CDK6, CDKN1A, CDKN2A, CEBPG, CHD8, CHEK1, CNOT8, COL15A1, COL1A1, COL1A2, COL3A1, COL4A1, COL4A2, COL4A5, COL5A1, COL5A2, COL5A3, COL8A1, CREB1, CRTAP, CTNNB1, CYP1A1, DDX19B, DICER1, DIO3, DLL1, DNMT1, DNMT3A, DNMT3B, DUSP1, DUSP2, E2F3, E2F5, EGFR, ELAVL1, EOMES, ERBB2, ERBB3, FBN1, FGF2, FGFR1, FNDC3B, FOXP1, GAS7, GMFB, GPR160, GPR37, GSS, H3-3A/H3-3B, HDAC1, HDAC4, HK2, ID1, ID2, ID3, IGFBP3, IGFBP5, IKZF4, IL1RN, JAG1, JARID2, KLF4, KRAS, KRT19, LAMC1, LEF1, LIN28A, LIPA, LOXL2, LOXL4, LRP6, MAN1A1, MAP2K1, MAP2K7, MAPK7, MAZ, MCL1, MDM2, MET, mir-9, MLF1, MYB, MYBL2, MYC, MYCN, MYD88, NASP, NAV3, NID1, NID2, NOTCH1, NOTCH2, NR1I2, OSBPL9, PCDHB10, PDGFA, PDGFB, PERP, PIK3R1, PLEC, PLK1, PPM1D, PPP2R1A, PPT2, PRC1, PTEN, PURA, RABL6, RERE, RPS6KA5, SCD, SGPL1, SH3BP4, SHROOM2, SIAH1, SIRT1, SMAD3, SMO, SP1, SPARC, SRSF10, ST18, TAGLN, TBX21, TCL1A, TDG, TENM2, TET1, TGFB3, TGFBR1, TGFBR2, THBS1, TNFAIP3, TOP2A, TP53, TRIM9, TRPS1, TSPAN8, TUBB2A, UGT2B17, UGT2B28, UVRAG, VCAN, VEGFA, WEE1, WNT1, WNT3, WNT3A, YY1, ZFP36L1, ZNF385A

## Data Availability

The data presented in this study are available within the article and its Supplementary Materials. Raw NanoString nCounter counts and digital PCR data are available from the corresponding author on reasonable request.

## Supplementary Materials

The following supporting information can be downloaded at: https://www.mdpi.com/article/10.3390/antiox15101311/s1, Supplementary Data S1: Normalized microRNA data; Figure S1: Per-donor IC_50_ concentration–response curves for 2D versus 3D conditions. Table S1: NDRI donor information including age, cause of death, comorbidities, ischemia time, and storage time; Supplementary Data S2: miRNA run data.
