# Supplementary material for: Differential Susceptibility to Heavy-Metal–Induced Injury in a Human Testis Tissue Equivalent (hTTE): Implications for Oxidative Stress
Source: Antioxidants (Basel). 2026 Oct 12;15(10):1311. doi: 10.3390/antiox15101311 (PMC13701779; doi:10.3390/antiox15101311)
Supplement: Supplementary file 1 [file antioxidants-15-01311-s001.zip › Figure S1.pdf]

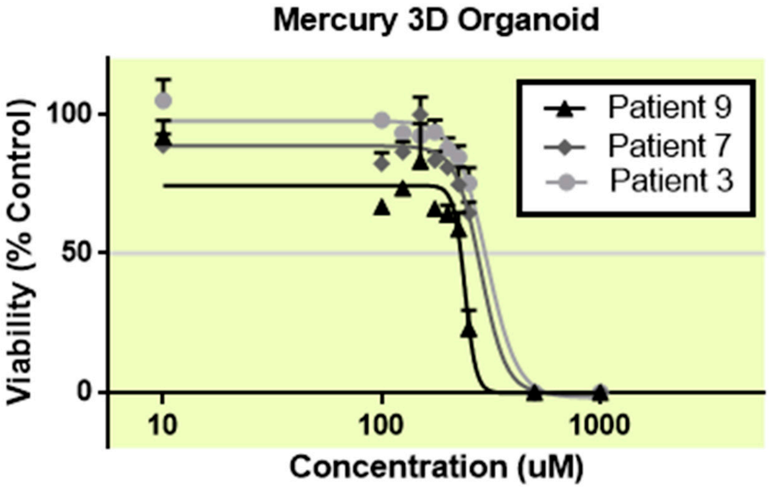
Mercury 3D Organoid
Viability (% Control)
100
50
0
10
100
1000
Concentration (uM)
Patient 9
Patient 7
Patient 3

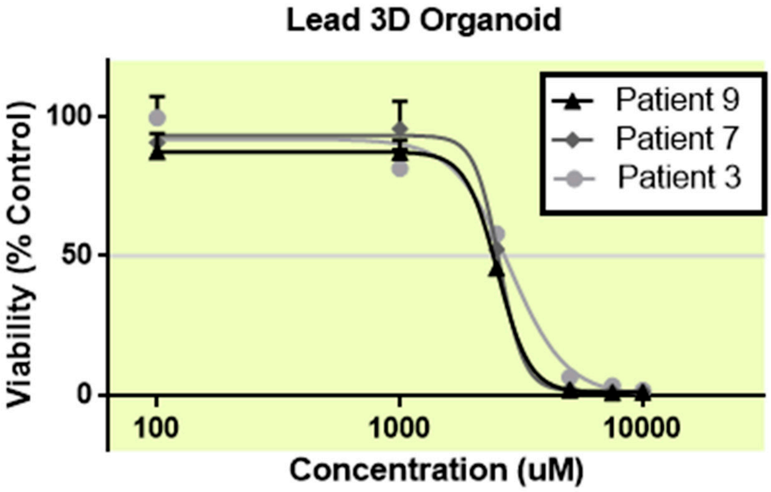
Lead 3D Organoid
Viability (% Control)
100
50
0
100
1000
10000
Concentration (uM)
Patient 9
Patient 7
Patient 3

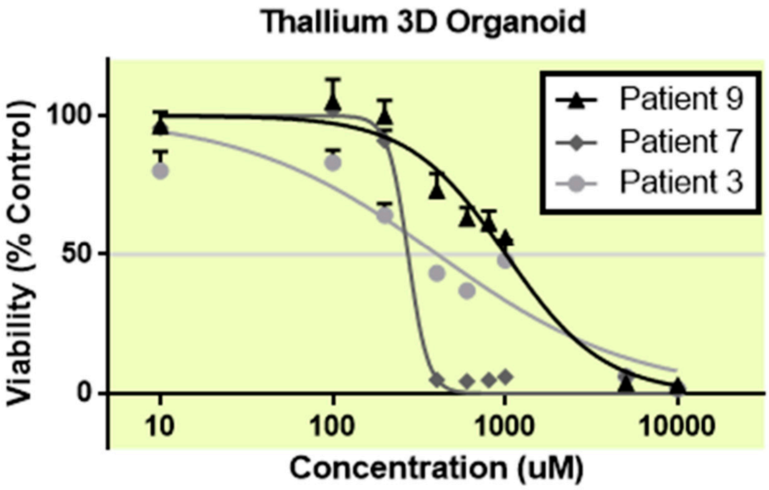
Thallium 3D Organoid
Viability (% Control)
100
50
0
10
100
1000
10000
Concentration (uM)
Patient 9
Patient 7
Patient 3

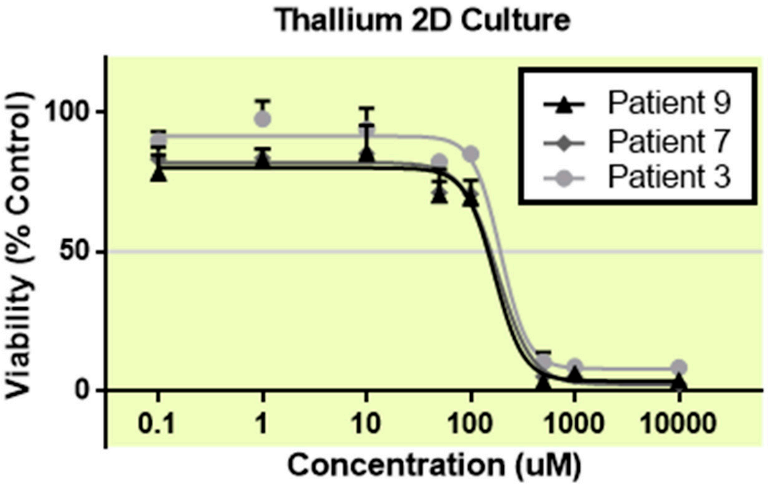
Thallium 2D Culture
Viability (% Control)
100
50
0
0.1
1
10
100
1000
10000
Concentration (uM)
Patient 9
Patient 7
Patient 3

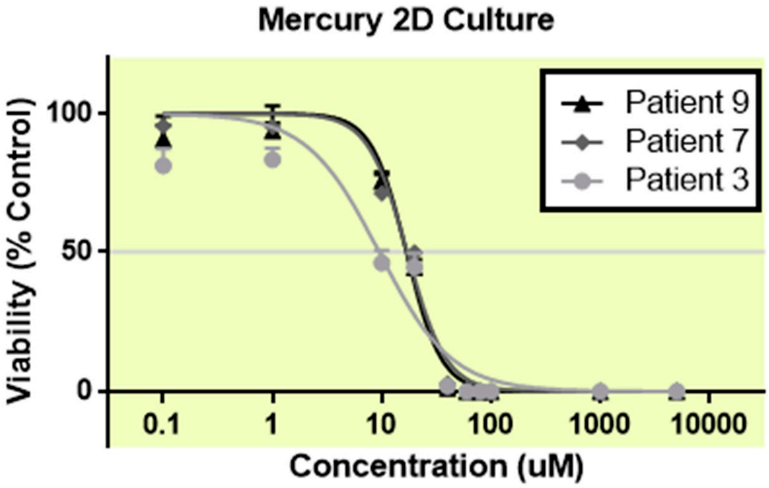
Mercury 2D Culture
Viability (% Control)
100
50
0
0.1
1
10
100
1000
10000
Concentration (uM)
Patient 9
Patient 7
Patient 3

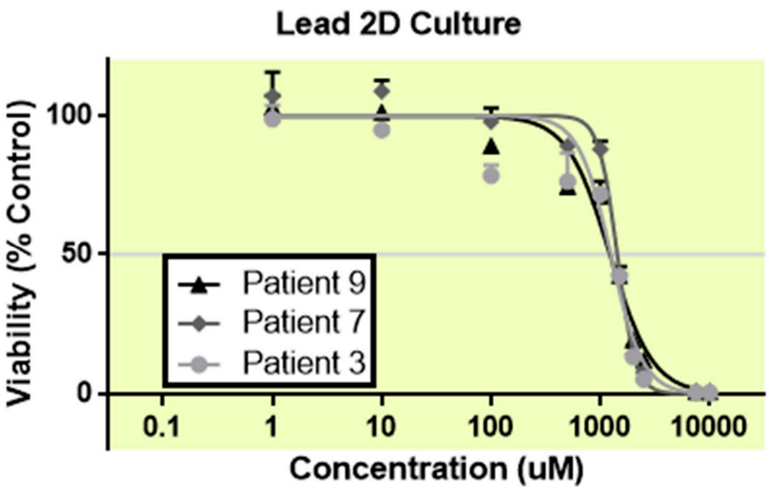
Lead 2D Culture
Viability (% Control)
100
50
0
Patient 9
Patient 7
Patient 3
0.1
1
10
100
1000
10000
Concentration (uM)

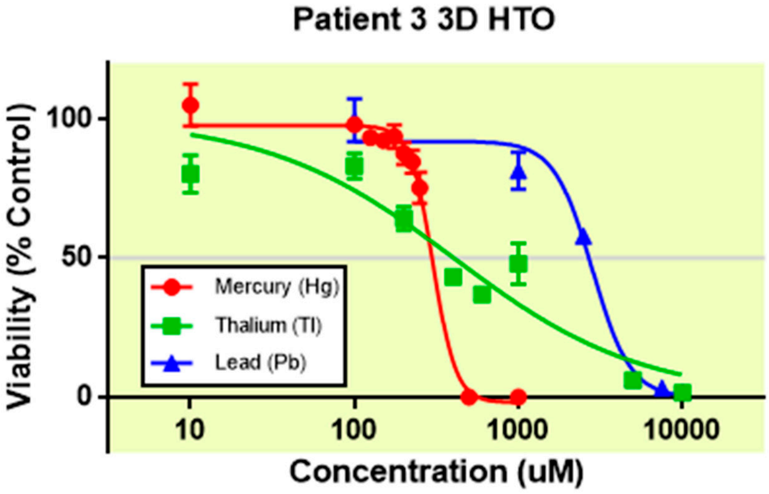
Patient 3 3D HTO
Viability (% Control)
100
50
0
Mercury (Hg)
Thalium (Tl)
Lead (Pb)
10
100
1000
10000
Concentration (uM)

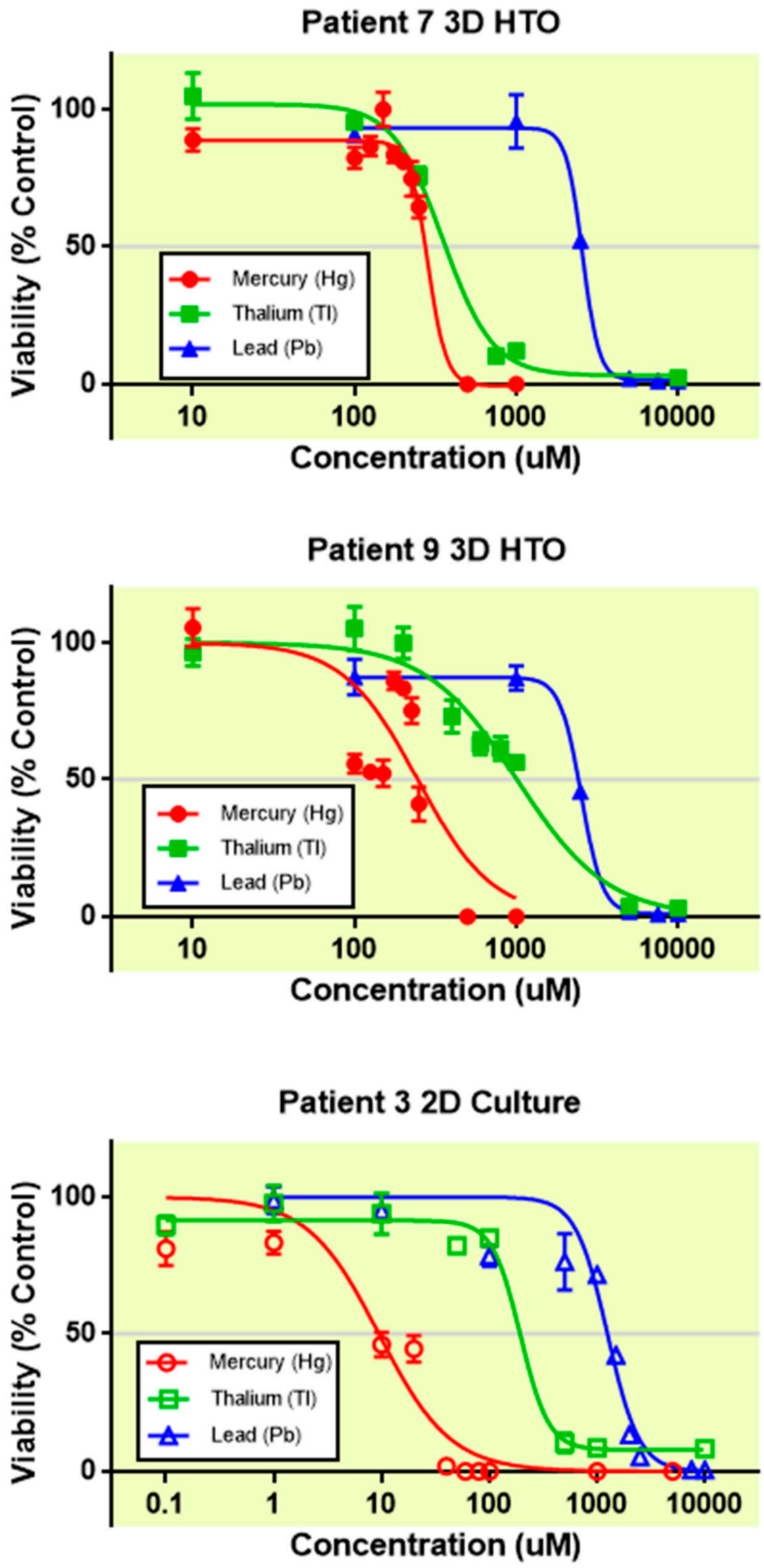
Patient 7 3D HTO
Viability (% Control)
100
50
0
Mercury (Hg)
Thalium (Tl)
Lead (Pb)
10
100
1000
10000
Concentration (uM)
Patient 9 3D HTO
Viability (% Control)
100
50
0
Mercury (Hg)
Thalium (Tl)
Lead (Pb)
10
100
1000
10000
Concentration (uM)
Patient 3 2D Culture
Viability (% Control)
100
50
0
Mercury (Hg)
Thalium (Tl)
Lead (Pb)
0.1
1
10
100
1000
10000
Concentration (uM)

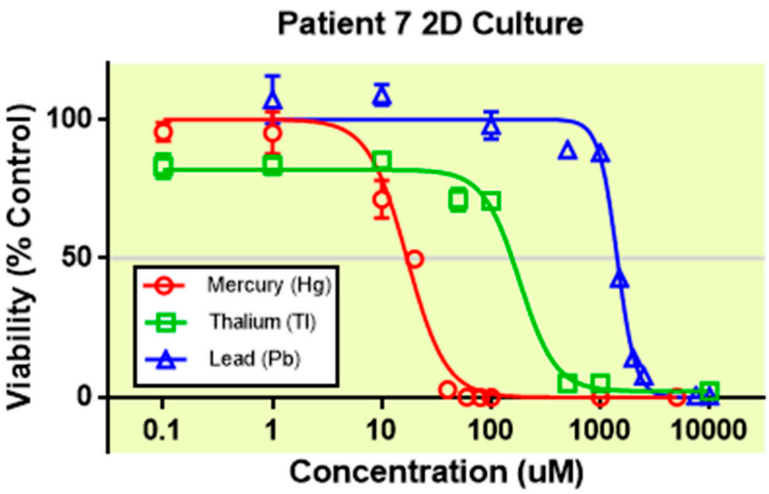
Patient 7 2D Culture
Viability (% Control)
100
50
0
0.1
1
10
100
1000
10000
Concentration (uM)
Mercury (Hg)
Thalium (Tl)
Lead (Pb)

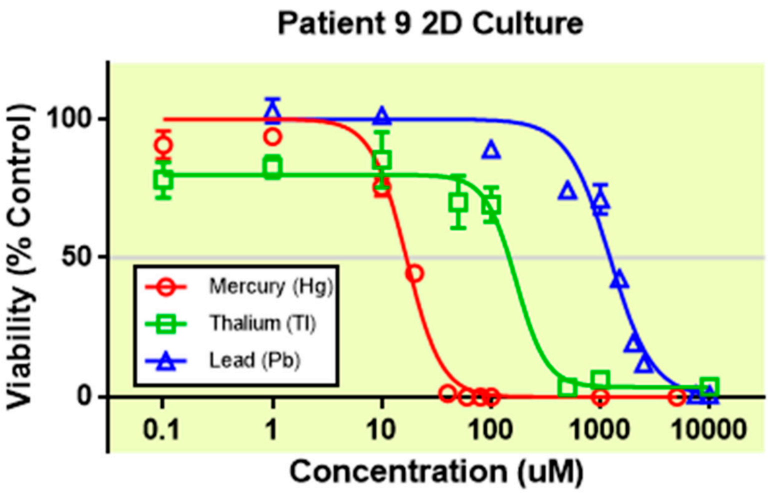
Patient 9 2D Culture
Viability (% Control)
100
50
0
0.1
1
10
100
1000
10000
Concentration (uM)
Mercury (Hg)
Thalium (Tl)
Lead (Pb)

Figure IC50

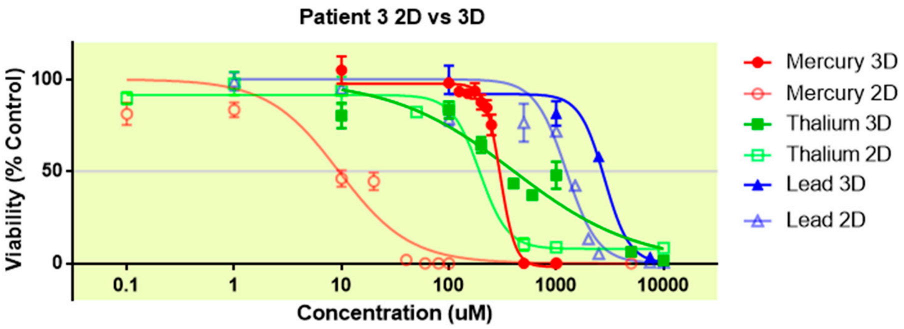
Patient 3 2D vs 3D
Viability (% Control)
100
50
0
0.1
1
10
100
1000
10000
Concentration (uM)
Mercury 3D
Mercury 2D
Thalium 3D
Thalium 2D
Lead 3D
Lead 2D

A

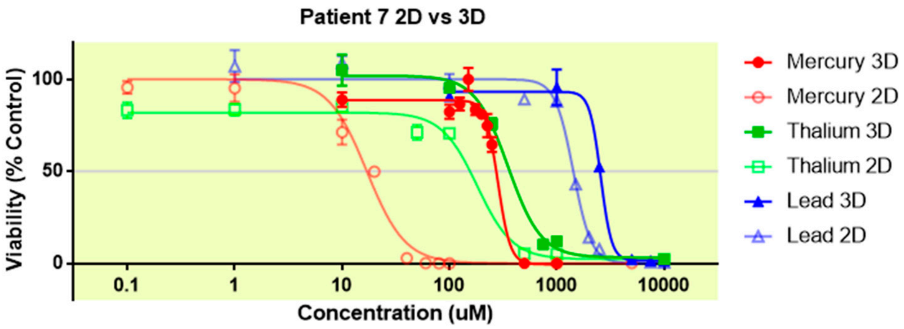
Patient 7 2D vs 3D
Viability (% Control)
100
50
0
0.1
1
10
100
1000
10000
Concentration (uM)
Mercury 3D
Mercury 2D
Thalium 3D
Thalium 2D
Lead 3D
Lead 2D

B

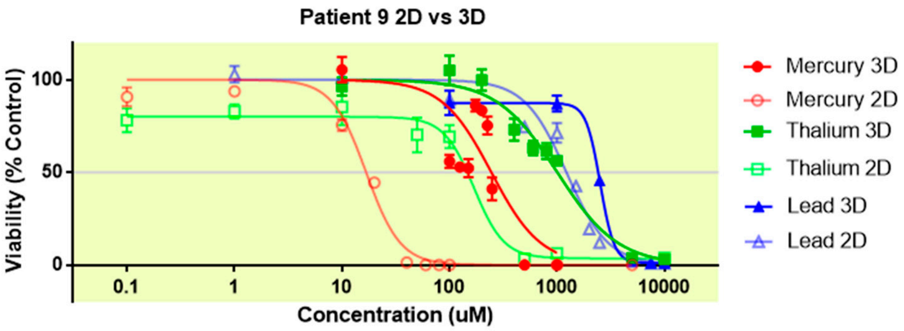
Patient 9 2D vs 3D
Viability (% Control)
100
50
0
0.1
1
10
100
1000
10000
Concentration (uM)
Mercury 3D
Mercury 2D
Thalium 3D
Thalium 2D
Lead 3D
Lead 2D

C

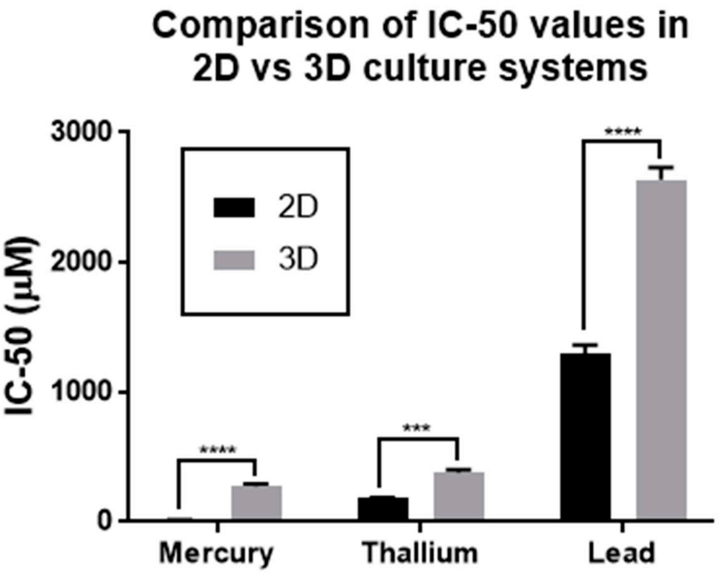

D

Toxic Agent	2D Mean (SD)	3D Mean (SD
**Mercury**	14.43 (4.31)	276.9 (28.5)
**Thallium**	181.33 (12.451)	378.15 (36.98)
**Lead**	1299.67 (110.726)	2638.33 (163.02)
