# Supplementary material for: Differential Susceptibility to Heavy-Metal–Induced Injury in a Human Testis Tissue Equivalent (hTTE): Implications for Oxidative Stress
Source: Antioxidants (Basel). 2026 Oct 12;15(10):1311. doi: 10.3390/antiox15101311 (PMC13701779; doi:10.3390/antiox15101311)
Supplement: Supplementary file 1 [file antioxidants-15-01311-s001.zip › Table S1 .pdf]

**Parameter**	**Patient 3 (NDRI 0068614)**	**Patient 7 (NDRI ND00324)**	**Patient 9 (NDRI ND01232)**
Age (years)	25	18	56
Sex	Male	Male	Male
Race code (A/R/S)	Black	Hispanic	Caucasian
Cause of death	Cardiac arrest (admitted with altered mental status/coma, possible pill ingestion, seizure → PEA; urine tox positive for opiates)	Gunshot wound to head → brain death (no surgical intervention)	Anoxia secondary to cardiac arrest (witnessed arrest, bystander CPR)
Height / Weight	71 in / 299 lb (~136 kg)	70 in / 80 kg	70 in / 220 lb (~100 kg)
Tissue / status	Testis, normal, fresh	Testes (bilateral), normal, fresh	Testes (bilateral), normal, fresh
Preservation / media	Fresh; DMEM + antibiotics	Fresh	Fresh
Diabetes	No	No	No
Tobacco	No	Yes (½ ppd × 2 yr)	Yes (1 ppd × 5 yr, current)
Alcohol	Yes	Yes	Social, quit 7 yr prior
Drugs	Opiates on tox screen	Marijuana	No
Relevant PMH	Hyperlipidemia, hypertension	None reported	HTN, CAD, COPD, CABG ×4, AICD/pacer, childhood asthma
Ventilator	Yes (since 5/28/2012)	Yes (3 days)	Yes (4 days)
Ischemia/timing	~7.9 h postmortem to recovery; recovery duration 01:22; death 6/6/2012 17:28 → recovery complete 6/7/2012 02:37	Heart-beating (brain-dead) donor maintained on ventilator; death declared 2/20/2013 15:15 → cross-clamp 2/22/2013 04:35	Warm ischemia time 23 min; death 6/9/2013 17:27, cross-clamp 6/9/2013 17:34; shipped same day
Procurement to shipping	6/7/2012 → 6/7/2012	2/21/2013 → 2/22/2013	6/9/2013 → 6/9/2013
